# Radon measurement and age-independent effective dose attributed to ingestion of bottled water in Iran: sensitivity analysis

**DOI:** 10.1038/s41598-023-39679-1

**Published:** 2023-08-05

**Authors:** Mina Pourshabanian, Simin Nasseri, Ramin Nabizadeh Nodehi, Sara Sadat Hosseini, Amir Hossein Mahvi

**Affiliations:** 1 Department of Environmental Health Engineering, School of Public Health, Tehran University of Medical Sciences, Tehran, Iran; 2 Center for Solid Waste Research, Institute for Environmental Research, Tehran University of Medical Science, Tehran, Iran

**Keywords:** Environmental sciences, Risk factors

## Abstract

A comprehensive study was made to measure the radon concentration in bottled water available in Iran market. The 222Rn concentration in 70 bottled water samples were measured by the sniffing mode technique and RTM 1688-2 (SARAD, Germany) in immediate sampling time and 3 months later for determination of radon decay. The measured radon concentration ranged from 0.003 to 0.618 Bq L^−1^ in bottled water samples, which were much lower than the recommended value for radon in drinking water by WHO (100 Bq L^−1^) and United states environmental protection agency (USEPA) (11.1 Bq L^−1^). The annual effective dose of 222Rn due to ingestion bottled water was also evaluated in this research. The mean annual effective dose due to ingestion of radon in bottled water for adults, children, and infants were estimated to vary from 5.30 × 10^−4^ mSv^−1^, 4.90 × 10^−4^ mSv^−1^, and 2.15 × 10^−4^ mSv^−1^, respectively. Overall, this study indicated that the Iranian people receive no significant radiological risk due to exposure to radon concentration in bottled water brands common consumed in Iranian market.

## Introduction

The environmental sources, especially groundwater drawn from granitic and metamorphic rocks are considered as the primary source of radioactivity^1,2^. Radioactive materials are commonly introduced into the environment via naturally occurring sources and man-made or artificial radionuclide fallouts^3,4^. The naturally occurring sources include the 238U and its daughter’s 226Ra and 222Rn, commonly observed in water resources, rocks and soil^4,5^. However, artificial radionuclide fallouts caused by accidents and nuclear explosion can contaminate the water resources and environment which human live^6,7^. Radon (222Rn), as the heaviest noble gas, which is produced via alpha decay of 238U is naturally ubiquitous in the environments; 222Rn alone comprises 50% of total natural radiation^8,9^. Radon gas is derived from soil, rock, and sediments and commonly observed in the groundwater due to high solubility (510 cm^3^ L^−1^) and density (9.73 g L^−1^)^10–13^. 222Rn is recognized as the colorless, tasteless, and odorless radioactive gas with half-life of 3.82 days^14,15^. Radon is characterized with long-term half-life and capability of alpha particle emission, causing lung, blood and gastrointestinal cancer in humans during long term exposure^5,16^. The inhalation and ingestion are the main pathways of exposure to 222Rn; the occurrence of high levels of this carcinogenic agent in indoor air and groundwater resources give rise to human internal exposure^16–19^. 222Rn exposure, as the second risk factor for lung cancer can also damage DNA, penetrate the stomach and move through the human body via the bloodstream^5,8^. World health organization (WHO) and United states environmental protection agency (USEPA) have recommended the values of 100 BqL^−1^ and 11.1 BqL^−1^, respectively, as the maximum contamination level in water resources^20,21^. Furthermore, 0.1 mSv^−1^ has been recommended by WHO as annual effective dose induced by exposure to 222Rn in drinking water^22,23^. The quantification of radioactivity in drinking water is vital for public health risk and exposure level of population and annual effective radiation doses^12,24^. Bottled water is considered as the major source of drinking water over the world, particularly in countries with hot and dry climates^3^. In recent year, bottled water contained in polyethylene in polyethylene terephthalate (PET) have drawn great attention and countries continue to produce bottled water in a large-scale application^25^. For instance, according to world water report, the average annual consumption of United Arab Emirates (UAE) has increased 260 L per person in 2006 Worldwater.org (http://www.worldwater.org/). Due to the fact that consumption of bottled water have increased worldwide, continuous monitoring of radon concentration in bottled water can facilitate the issues and concerns about the water supply. Recently, several studies have focused on radon levels in various drinking water such as bottled water, groundwater and surface water over the world^26–30^. In case of Iran, some studies have been focused on radon concentration in water resources including spring, wells, Qanat and tap water^5^. However, to best of our knowledge, no information are available on radon concentration in Bottled water. This study could be regarded as the first attempt to comprehensively monitor the radon concentration in Iran and estimate the annual effective dose by Monte Carlo stimulation technique.

## Materials and methods

### Sampling

This cross-sectional study was performed in 2019 to comprehensively monitor radon concentration in bottled water in the Iranian market. A total of 70 different bottled water brands mostly consumed by Iranian people were collected from different provincial areas of Iran. The bottled waters were transferred to the laboratory, each bottle of water had specific code. The radon measurements were performed twice; immediately after collection of bottled water and three months later in order to determine radon decay after production. Special care was taken to avoid the exposure of water samples in bottled water with air and kept at 25 °C.

### Measurement of radon concentration in water

The concentration of 222Rn in the bottled water was measured by the slow-mode technique and RTM 1688-2 (SARAD, Germany) using radon gas extraction from the solution^31^. A brief description of radon measurement procedure is described here. Generally, the Radon (222Rn) gas concentration is determined by the short living daughter products, generated by the Radon decay inside a measurement chamber. In addition, the measurement of radon activity concentration in the water sample is based on the equilibrium state between the Radon air and water activity concentrations which takes place within a sealed system after a certain time (1 h). The bubbling flask containing 500 mL of bottled water at 25 °C was connected to RTM 1688-2 device. The bubbling Flask enables release of radon gas form the water sample, which is directed to a closed loop. The released volume air circulates the loop by the internal pump of Radon monitor. Immediately after the radon decay, the remaining Po-218 nuclei becomes charged positively for a short period; some shell electrons are scattered away by the emitted alpha particle. At the same time, the semiconductor detector shows the ions collected by the electrical field force; the numbers of collected Po-218 ions is proportional to the Radon gas concentration inside the chamber. Generally, the total radon activity within the system is calculated as a constant over the measurement period and the relationship can be stated as per the formula^31^:

1 $${\text{A}}\left( {{\text{Water}}} \right) \, = {\text{ A1}}\left( {{\text{Water}}} \right) \, + {\text{ A }}\left( {{\text{Air}}} \right),$$

with: A(Water) = Total activity of the water sample before the de-gassing, A1 (Water) = Remaining activity of the water sample after the de-gassing, A(Air) = activity in the air volume.

In addition, the radon concentrations were reported with 95% confidence interval and BqL^−1^. The pH, and conductivity were measured for different bottled brands.

### Estimation of annual effective doses (AED)

The exposure to radon concentration in drinking water in long-term period contribute to increased radiation dose exposure on stomach^9^. The average annual effective dose (Ed, mSvy^−1^) due to exposure of radon following ingestion the bottled water rich in Radon was estimated using the procedure and equation recommended by UNSCEAR^5,12^:

2 $${\text{E}}_{{\text{d}}} = {\text{ A}}_{{\text{c}}} \times {\text{ q }} \times {\text{ C}}_{{\text{f}}} \times {\text{T}},$$

where, A_c_ refers to radon activity concentration in bottled water samples (BqL^−1^), q represents the daily consumption rate (Lday^−1^), Cf is the dose coefficient (mSvBq^−1^) for different groups of ages, and T is ingestion period (365 days).

It is important to note that there is no valid and official data on consumption rate of bottled drinking water for Iran, therefore, we have adopted data reported United Nations Scientific Committee on the Effects of Atomic Radiation^16^ for different group: infants (150 Lyr^−1^), children (350 Lyr^−1^), and adults (730 Lyr^−1^). The dose coefficient (Cf) for each age group was obtained from “International Basic Safety Standards for Protection against Ionizing Radiation and for Safety of Radiation Sources”^32^: Adults (18 × 10^−6^ mSv/Bq^−1^**)** and Children (35 × 10^−6^ mSvBq^−1^).

### Monte Carlo simulation technique

The uncertainty and variability of the parameters considered in the equation of risk index were calculated with Monte-Carlo simulation technique and 10,000 iterations in the Oracle® Crystal Ball software (1.1.2.4.850 version). This technique selects the values of the parameters in the specified range proportional to the distribution of each variable, and then calculates the risk level. This process is repeated several times and calculates the average, minimum, maximum, standard deviation, different percentile values, and some other statistical indices as the final result. These repetitions remove the uncertainty and variability of the parameter values. Therefore, the results are more reliable and more valuable than the obtained results with constant values of input parameters. In order to determine the contribution of input variables in the predicted value of the risk index, sensitivity analysis was conducted. On the other hand, the sensitivity analysis indicated how much influential factors can change the response variable, annual effective dose for different groups of ages.

## Results and discussion

### Radon activity concentration in bottled water

A total of 70 bottled water brands that were commercially available in Iran market and different provinces were collected and analyzed for 222Ra concentration (BqL^−1^). Table 1 presents the mean radon concentration in bottled water samples immediately collected from Iran market and its concentration after 3 months storage in 25 °C. As seen in Table 1, the radon concentration varies from 0.003 to 0.618 BqL^−1^. The highest and lowest 222Rn concentration value were observed in Codes 35 and 49, respectively. In addition, the mean value of radon measurement in the initial stage was calculated to be 0.040 BqL^−1^, which is much lower the recommended values of 222Rn in drinking water USEPA (11.1 BqL^−1^) and WHO (10 BqL^−1^)^33,34^. The decay of 222Rn concentration in bottled water samples storage at environment temperature within 3 months were calculated to be 0.001 to 0.593 BqL^−1^. Generally, the 222Rn concentrations in all bottled water samples were measured to be lower than the recommended level and are acceptable for drinking water in Iran. Abojassim et al. surveyed the radon concentration in bottled water in Iraq market. They reported that the mean radon concentration in drinking bottled water were 0.11256 BqL^−1^ (0.0354–0.248 BqL^−1^)^35^. In addition, Turhan et al. reported that the average gross alpha and beta radioactivity concentrations in bottled water in Turkey were 21 ± 5 m BqL^−1^ and 59 ± 12 mBqL^−1^, respectively^2^. In a systematic review made in Iran, Keramati et al. reported that radon concentration in drinking waters were lower the guideline value except springs^5^, which are in consistent with our study. The less solubility and short-lived period of radon leads to release of this radioactive gas to atmosphere from surface water. That is the most likely explanation of lower observed radon concentration in tap water compared to water taken from depths^36^. In addition, Table S1 lists the pH and EC of bottled water samples in initial stage and measurement after three months.

**Table 1:** Radon concentration in different bottled water samples available in Iran market at start and after three months.

Code	Initial Ra-22 concentration (BqL^−1^)	Decay Ra-22 concentration (BqL^−1^)	Code	Initial Ra-22 concentration (BqL^−1^)	Decay Ra-22 concentration (BqL^−1^)
1	0.008 ± 0.001	0.005	36	0.008 ± 0.001	0.004
2	0.003 ± 0.001	0.002	37	0.003 ± 0.001	0.001
3	0.006 ± 0.001	0.004	38	0.008 ± 0.001	0.006
4	0.008 ± 0.001	0.003	39	0.278 ± 0.011	0.039
5	0.003 ± 0.001	0.002	40	0.017 ± 0.005	0.006
6	0.014 ± 0.002	0.011	41	0.302 ± 0.004	0.041
7	0.007 ± 0.002	0.005	42	0.092 ± 0.010	0.023
8	0.005 ± 0.001	0.002	43	0.003 ± 0.001	0.001
9	0.006 ± 0.002	0.004	44	0.003 ± 0.001	0.002
10	0.038 ± 0.004	0.021	45	0.011 ± 0.001	0.005
11	0.003 ± 0.001	0.001	46	0.003 ± 0.001	0.002
12	0.006 ± 0.001	0.004	47	0.038 ± 0.008	0.015
13	0.003 ± 0.001	0.002	48	0.023 ± 0.004	0.014
14	0.008 ± 0.001	0.005	49	0.003 ± 0.001	0.002
15	0.005 ± 0.001	0.002	50	0.01 ± 0.001	0.006
16	0.385 ± 0.006	0.015	51	0.006 ± 0.001	0.002
17	0.003 ± 0.001	0.002	52	0.014 ± 0.002	0.005
18	0.309 ± 0.009	0.006	53	0.008 ± 0.002	0.004
19	0.015 ± 0.003	0.008	54	0.011 ± 0.001	0.005
20	0.008 ± 0.001	0.006	55	0.016 ± 0.001	0.006
21	0.176 ± 0.003	0.032	56	0.008 ± 0.001	0.005
22	0.02 ± 0.006	0.012	57	0.006 ± 0.002	0.002
23	0.008 ± 0.001	0.06	58	0.005 ± 0.002	0.002
24	0.003 ± 0.001	0.001	59	0.009 ± 0.001	0.005
25	0.032 ± 0.002	0.017	60	0.015 ± 0.003	0.008
26	0.003 ± 0.001	0.001	61	0.005 ± 0.001	0.001
27	0.085 ± 0.004	0.014	62	0.015 ± 0.001	0.006
28	0.003 ± 0.001	0.002	63	0.007 ± 0.001	0.003
29	0.009 ± 0.001	0.007	64	0.015 ± 0.001	0.009
30	0.008 ± 0.001	0.004	65	0.019 ± 0.004	0.005
31	0.017 ± 0.003	0.012	66	0.009 ± 0.001	0.006
32	0.02 ± 0.004	0.011	67	0.008 ± 0.001	0.003
33	0.005 ± 0.001	0.004	68	0.005 ± 0.001	0.002
34	0.006 ± 0.001	0.002	69	0.017 ± 0.001	0.008
35	0.618 ± 0.001	0.025	70	0.012 ± 0.002	0.005

### Associated age specific annual effective dose estimation

**Figure 1:**
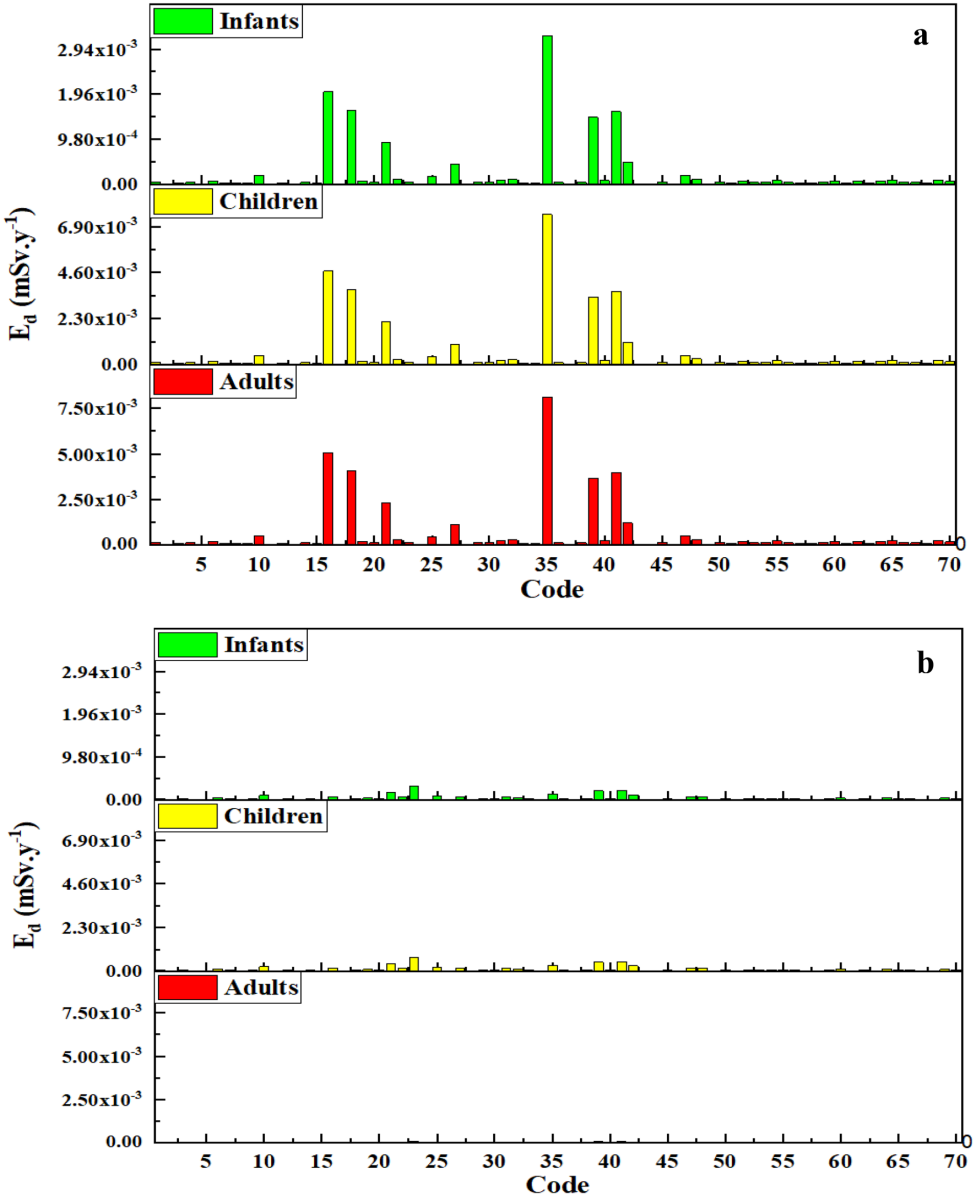
The calculated annual effective doses of radon in bottled water samples in initial stage (**a**), and stored at 25 °C for 3 months (**b**).

The calculation of annual effective dose can provide good interpretation of health risks in exposure to pollutant^37^. The annual effective doses of radon in bottled water samples due to ingestion were calculated for different groups of ages (viz., infant, child and adult) using the parameters presented in Table 2. Figure 1a,b reveals the annual mean effective doses of radon in bottled water consumed by Iranian people for different groups of ages. Figure 1a shows the corresponding annual effective dose of radon in the bottled water samples in the immediate and initial stage. For infants, the Ed value varies from 1.57 × 10^−5^ to 3.20 × 10^−3^ mSvy^−1^ with average value of 2.15 × 10^−4^ mSvy^−1^. In case of children group of ages, the Ed were calculated to be in range of 3.64 × 10^−5^–7.50 × 10^−3^ mSvy^−1^ with average value of 4.90 × 10^−4^ mSvy^−1^. As for adults group, the annual effective dose was estimated to be between 3.94 × 10^−5^ and 8.10 × 10^−3^ mSvy^−1^ with average value of 5.30 × 10^−4^ mSv^−1^. Based on these results, the Ed for adults groups was found to be the highest followed by infants and children. Therefore, the adults are considered to be the most critical population group for consuming bottled water in Iran. As the dose conversion coefficient in infants and children are twice that of adults group, the annual consumption rate of bottled water is most likely influencing parameter on higher Ed in adults group. According to result obtained from the measurement of radon in bottled water brands studied in the present research, we found that all annual effective dose of radon in different groups of ages is much lower the maximum annual reference dose recommended by WHO and UNSCEAR (0.1 mSvy^−1^)^22,23^. The similar results with Ed lower than the normal limits of the world (i.e. 1 mSv^−1^) were previously reported in studies focused on radon in water resources^3,8,35^.

**Table 2:** Parameters used in calculating annual effective doses of radon in bottled water samples.

Parameter	Age group (years)			Distribution	Refs.
	Infants	Children	Adults		
Water ingestion (Ld^−1^)	0.45 ± 0.12	1.12 ± 0.27	1.23 ± 0.27	Log normal	^38^
Radon concentration (BqL^−1^)	Initial: 0.03 ± 0.09; storaged: 0.01 ± 0.01			Log normal	This study
Dose coefficient (mSvBq^−1^)	35 × 10^−6^		18 × 10^−6^	–	^12^
Exposure frequency (day)	0–365	0–365	0–365	Uniform	This study

In addition, the annual effective doses of radon in bottled water samples stored at 25 °C for three months are shown in Fig. 1b. As shown in Fig. 1b, the Ed for adults group of age were calculated to be in range of 1.31 × 10^−6^–7.88 × 10^−5^ mSvy^−1^ with average value of 1.07 × 10^−5^ mSvy^−1^. In case of children group, the Ed values varies from 1.21 × 10^−5^ to 7.28 × 10^−4^ mSvy^−1^ with average value of 9.93 × 10^−5^ mSvy^−1^. As for infants, this value was estimated to be between 5.24 × 10^−6^ and 3.14 × 10^−4^ mSvy^−1^ with average value of 4.28 × 10^−5^ mSvy^−1^. Generally, the Ed estimated for radon concentration in storage bottled water samples are significantly lower than those of initial samples. Overall, for the investigated radon concentration in bottled water, it can be concluded that the Iranian people receive no significant radiological risk due to exposure to radon concentration in bottled water brands commonly observed.

### Sensitivity analysis

Sensitivity analysis using Monte-Carlo simulation was performed to investigate how variability of the outputs can be apportioned quantitatively to different sources of variability in the inputs. In addition, the Monte-Carlo simulation technique and sensitivity analysis aid the authorities to understand which influential factors affect more on annual effective dose.

**Figure 2:**
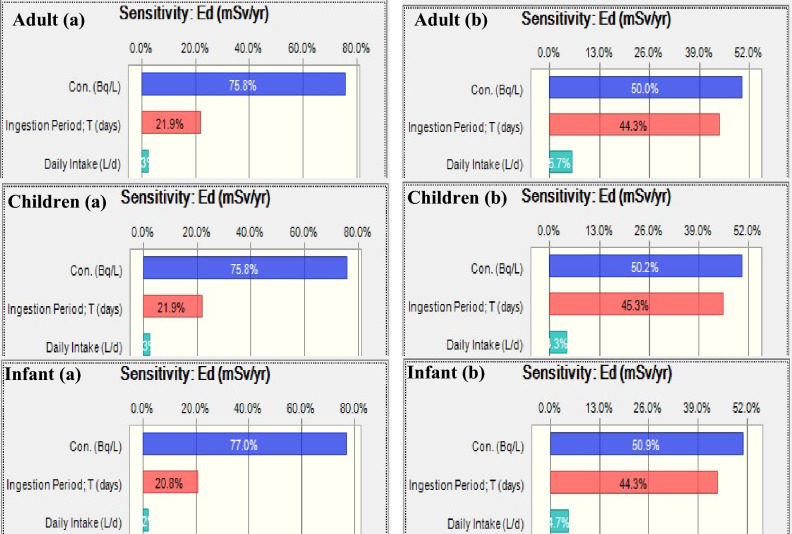
Sensitivity analysis and the contribution of influencing parameters on annual effective doses of radon in bottled water. (**a**) (Initial), (**b**) (storaged).

The results for risk assessment through ingestion of bottled water with radon show that in both measurements (initial and after storage), radon concentration has the highest contribution to variation of this index (see Fig. 2). As seen from Fig. 2, the contribution of radon concentrations in initial stage ranged from 75.8 to 77.0%; these values were estimated to be 50.0 to 50.9% for bottled water samples storaged at temperature 25 °C.

This highlights the importance of radon concentration in exposure to bottled water and water sources rich in radon. Therefore, this parameter should be monitored continuously by the authorities and preventive measurements are suggested to take radon concentration in water sources as priority.

## Conclusion

The present study represents one of first attempt to comprehensively monitor the radon activity concentration in bottled water available in Iran market. Our conclusions are based on radon concentration in 70 bottled water brand common consumed by Iranian people: (1) the measured radon concentration in bottled water sample (0.003–0.618 BqL^−1^) were much lower than the values recommended by WHO and UNCERP. (2) The annual effective doses of radon in bottled water were estimated to be lower than the valued recommended by WHO. (3) Overall, it can be concluded that the Iranian people receive no significant radiological risk due to exposure to radon concentration in bottled water brands commonly observed. However, the continuous monitoring of radon concentrations in different sources can aid authorities to provide mitigation action in order to minimize the health risk. In addition, the limitation of present study can be described such that there is no official consumption rate for bottled water in Iran, therefore, the authorities are suggested to measure this value to aid the definite annual effective doses of radon in bottled water.

### Supplementary Information

Supplementary Table S1.

## Data Availability

The datasets generated and analyzed during the current study available from the corresponding author on reasonable request.
