# Supplementary material for: Radon measurement and age-independent effective dose attributed to ingestion of bottled water in Iran: sensitivity analysis
Source: Sci Rep. 2023 Aug 5;13:12717. doi: 10.1038/s41598-023-39679-1 (PMC10404218; doi:10.1038/s41598-023-39679-1)
Supplement: Supplementary file 1 — Supplementary Table S1. [file 41598_2023_39679_MOESM1_ESM.docx]

**Table S1. pH and electrical conductivity (EC) measured in in different bottled water samples available in Iran market at start and after three months.**

| **Code** | **pH** | **EC (Sm^−1^)** | **Code** | **pH** | **EC (Sm^−1^)** |
| --- | --- | --- | --- | --- | --- |
| **1** | 8 | 396 | **1** | 8.04 | 390 |
| **2** | 8 | 367 | **2** | 7.31 | 363 |
| **3** | 7.5 | 98 | **3** | 7.08 | 97 |
| **4** | 7.5 | 213 | **4** | 7.05 | 215 |
| **5** | 7.1 | 348 | **5** | 6.67 | 352 |
| **6** | 7.2 | 189 | **6** | 6.98 | 190 |
| **7** | 7.7 | 172 | **7** | 6.92 | 174 |
| **8** | 8 | 432 | **8** | 8 | 434 |
| **9** | 8.1 | 351 | **9** | 7.9 | 351 |
| **10** | 7.1 | 98 | **10** | 6.89 | 97 |
| **11** | 7.5 | 202 | **11** | 7.57 | 201 |
| **12** | 8.1 | 306 | **12** | 8.2 | 305 |
| **13** | 8.2 | 347 | **13** | 8.1 | 348 |
| **14** | 7.5 | 424 | **14** | 8 | 420 |
| **15** | 7.5 | 275 | **15** | 7.4 | 277 |
| **16** | 7 | 146 | **16** | 7 | 149 |
| **17** | 8.1 | 235 | **17** | 8.2 | 234 |
| **18** | 8.2 | 539 | **18** | 8.5 | 533 |
| **19** | 7.35 | 299 | **19** | 7.2 | 298 |
| **20** | 7.5 | 332 | **20** | 7.9 | 331 |
| **21** | 7.1 | 338 | **21** | 7.1 | 399 |
| **22** | 8.1 | 341 | **22** | 8.2 | 343 |
| **23** | 7.5 | 221 | **23** | 7.9 | 221 |
| **24** | 7.1 | 244 | **24** | 6.9 | 240 |
| **25** | 7.8 | 339 | **25** | 8.2 | 329 |
| **26** | 7.5 | 247 | **26** | 7.4 | 238 |
| **27** | 8 | 370 | **27** | 8.3 | 364 |
| **28** | 7.2 | 277 | **28** | 7.1 | 273 |
| **29** | 8.2 | 517 | **29** | 8.4 | 515 |
| **30** | 8.1 | 419 | **30** | 8.3 | 420 |
| **31** | 7.5 | 476 | **31** | 7.6 | 476 |
| **32** | 8.1 | 182 | **32** | 8.2 | 181 |
| **33** | 7.2 | 283 | **33** | 7.3 | 288 |
| **34** | 7.5 | 275 | **34** | 7.9 | 279 |
| **35** | 7.1 | 385 | **35** | 7.3 | 388 |
| **36** | 7.1 | 271 | **36** | 6.6 | 270 |
| **37** | 7.2 | 165 | **37** | 7.3 | 163 |
| **38** | 8.5 | 378 | **38** | 8.8 | 379 |
| **39** | 8 | 336 | **39** | 8 | 334 |
| **40** | 8.1 | 452 | **40** | 8 | 451 |
| **41** | 7.5 | 303 | **41** | 7.6 | 302 |
| **42** | 7.5 | 236 | **42** | 7.7 | 235 |
| **43** | 7.5 | 104 | **43** | 7.7 | 103 |
| **44** | 7.4 | 340 | **44** | 7.8 | 336 |
| **45** | 7.5 | 582 | **45** | 7.5 | 580 |
| **46** | 8 | 361 | **46** | 8 | 352 |
| **47** | 7.6 | 281 | **47** | 7.9 | 282 |
| **48** | 7.5 | 372 | **48** | 7.9 | 370 |
| **49** | 7.6 | 323 | **49** | 7.8 | 312 |
| **50** | 7.5 | 371 | **50** | 8 | 374 |
| **51** | 7.5 | 261 | **51** | 8 | 365 |
| **52** | 7.6 | 364 | **52** | 7.7 | 365 |
| **53** | 7.6 | 202 | **53** | 8.2 | 203 |
| **54** | 8 | 356 | **54** | 8.6 | 349 |
| **55** | 8.1 | 245 | **55** | 8.5 | 238 |
| **56** | 7.7 | 372 | **56** | 8 | 373 |
| **57** | 7.8 | 255 | **57** | 8.1 | 253 |
| **58** | 7.2 | 181 | **58** | 7.3 | 180 |
| **59** | 8.01 | 382 | **59** | 7.9 | 380 |
| **60** | 7.5 | 321 | **60** | 8 | 320 |
| **61** | 7.4 | 431 | **61** | 7.6 | 435 |
| **62** | 7.6 | 237 | **62** | 7.7 | 238 |
| **63** | 8.1 | 549 | **63** | 8.2 | 553 |
| **64** | 7.6 | 173 | **64** | 7.9 | 174 |
| **65** | 7.9 | 205 | **65** | 8.1 | 204 |
| **66** | 7.5 | 345 | **66** | 7.67 | 346 |
| **67** | 8.1 | 317 | **67** | 8.22 | 315 |
| **68** | 7.7 | 449 | **68** | 7.89 | 451 |
| **69** | 7.8 | 491 | **69** | 7.89 | 493 |
| **70** | 7.9 | 418 | **70** | 7.94 | 417 |
